# Postoperative irradiation in patients with pT3-4N0 laryngeal cancer: results and prognostic factors

**DOI:** 10.1007/s00405-014-3333-7

**Published:** 2014-11-29

**Authors:** Tomasz Skóra, Jadwiga Nowak-Sadzikowska, Anna Mucha-Małecka, Bogumiła Szyszka-Charewicz, Jerzy Jakubowicz, Bogdan Gliński

**Affiliations:** Oncology Department, Maria Skłodowska-Curie Memorial Institute of Oncology, ul. Garncarska 11, 31-115 Kraków, Poland

**Keywords:** Laryngeal cancer, Postoperative radiotherapy, Total laryngectomy, Prognostic factors

## Abstract

Approximately 60 % of patients with locally advanced laryngeal cancer (LALC) treated primarily with surgery require adjuvant radiotherapy. In the available literature predominate series of patients were with pathologically confirmed node-positive status. Subgroups of pN0 patients with LALC are scarce. The aim of the study is to evaluate the efficacy of postoperative radiotherapy in patients with pathological stage T3-4N0M0 and identification of prognostic factors in this group. Between 1975 and 2005, 138 patients with squamous pT3-4N0 laryngeal cancer were irradiated postoperatively. Primary surgical treatment consisted of total laryngectomy and cervical lymphadenectomy. The median time between surgery and the implementation of radiotherapy was 56 days. The median total dose was 60 Gy (range 40–70 Gy). Five-year disease-free survival (DFS5) was achieved in 76 % of patients. Cancer recurrence was observed in 34 patients. In 28 (82 %) cases it was locoregional failure. DFS5 rates for pT3 and pT4 were 92 and 69 %, for margin status R0, R1 and R2 were 82, 72 and 67 %, respectively. The pharyngeal invasion was related to a decrease in DFS5 from 80 to 59 %. Postoperative irradiation in patients with pT3-4N0 LALC is an effective treatment method. The main reason of the failure is local recurrence. The following independent prognostic factors were identified in this group of patients: pT stage, surgical margin status and pharyngeal invasion.

## Introduction

The treatment of patients with locally advanced laryngeal cancer (LALC) includes three basic methods of therapy: surgery, radiotherapy and chemotherapy used alone or in combination in different time sequences. The effectiveness of definitive radiotherapy alone in LALC patients is controversial, 5-year survival rates range from 10 to 40 % [1, 2]. In the study by Mucha-Małecka and Składowski assessing 114 patients with pT4 laryngeal cancer treated with radiotherapy alone, 3-year rates for overall survival, disease-free survival and local control were 40, 35 and 42 %, respectively [3]. The introduction of concurrent chemoradiotherapy improved locoregional treatment efficacy and prolonged overall survival in approximately 60 % of LALC cases [4, 5]. Nowadays, according to different authors, 30–50 % of patients with LALC require primarily total laryngectomy with the various types of neck dissections. The range of neck lymphadenectomy depends on the specific clinical situation and experience of the operating centre. It is estimated that around 60 % of LALC patients initially treated with surgery require adjuvant radiation. The widely accepted indications for postoperative radiotherapy are: the cervical lymph nodes metastases, extracapsular extension, high-grade tumors, positive surgical margins, invasion beyond the anatomical boundaries of the larynx and subglottic extension. Postoperative radiation in patients with these adverse factors is considered a standard clinical practice [6–9]. In the published series predominate were node-positive cases. Subgroups of patients with pathologically negative regional lymph nodes, despite the extensively locally advanced tumor, are scarce [10–12]. Therefore, it seems reasonable to present our experience based on a larger number material relating to this group of patients. The aim of the study is to evaluate the efficacy of postoperative radiotherapy in LALC patients after total laryngectomy without cervical lymph node metastases (pT3-4N0M0) and prognostic factors identification in this group.

## Materials and methods

A total of 138 patients with pT3-4N0 squamous cell laryngeal cancer postoperatively irradiated between 1975 and 2005 at the Oncology Centre in Kraków, were retrospectively enrolled. The study group consisted of 132 men and 6 women. The median age was 57 years (34–89 years). All patients were initially treated with total laryngectomy and neck dissection. The type of lymphadenectomy was dependent on the particular clinical situation. In all cases, the presence of metastases in regional lymph nodes was excluded based on histopathological examination. In this period of time indications for adjuvant radiotherapy included: pT3 with unfavorable factors (extensive infiltration of surrounding tissues, positive surgical margins, subglottic tumor extension, poor differentiation of cancer) and all pT4 cases. The median interval between surgery and postoperative radiotherapy was 56 days (25–147 days). The total dose was between 45 and 70 Gy (median 60 Gy) in 2 Gy fractions. The endpoint of the study was the five-year disease-free survival (DFS5). It was defined as the time from the surgery to reappearance of locoregional disease, metastases or patient death. The prognostic power of analyzed parameters was assessed in relation to this value. The shortest period of follow-up was 7 years. In the analysis of the statistical differences between survival rates, the log-rank test by Peto was used. The influence of selected factors on the patient prognosis was assessed with Cox proportional hazards model [13, 14]. The effectiveness of radiotherapy was evaluated, depending on the parameters characterizing: patient (age, sex, performance status by Karnofsky Performance Score, haematocrit, hemoglobin), cancer (differentiation grade, subglottic extension, pT, adjacent anatomical structures invasion—the throat, the trachea) and selected treatment characteristics (surgical margins status, interval between surgery and postoperative radiotherapy, total dose, overall radiotherapy time).

## Results

The median follow-up was 76 months. The irradiation tolerance during the treatment was very good (in 96 % of patients the total dose and fractionation protocols were administered as planned). Severe late radiation-induced toxicity occurred in 3 patients. In 2 cases (1.4 %) pronounced skin and subcutaneous tissue fibrosis causing a significant multidirectional restriction of the neck movements, was noticed. In both patients entire cervical lymphatic system was treated with cobalt-60 radiation. In case of the one patient (0.7 %) tracheoesophageal fistula occurred 9 months after radiotherapy, successfully cured by surgery.

**Fig. 1:**
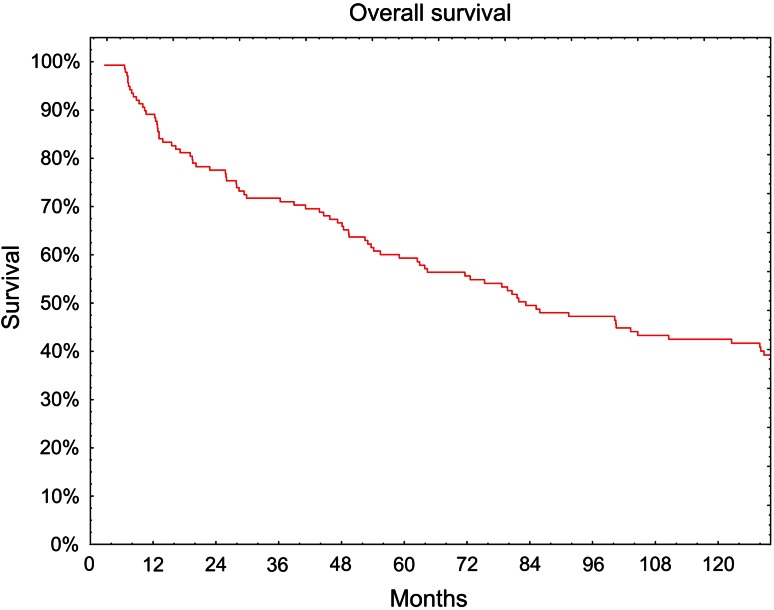
Overall survival (Kaplan–Meier curve)

**Fig. 2:**
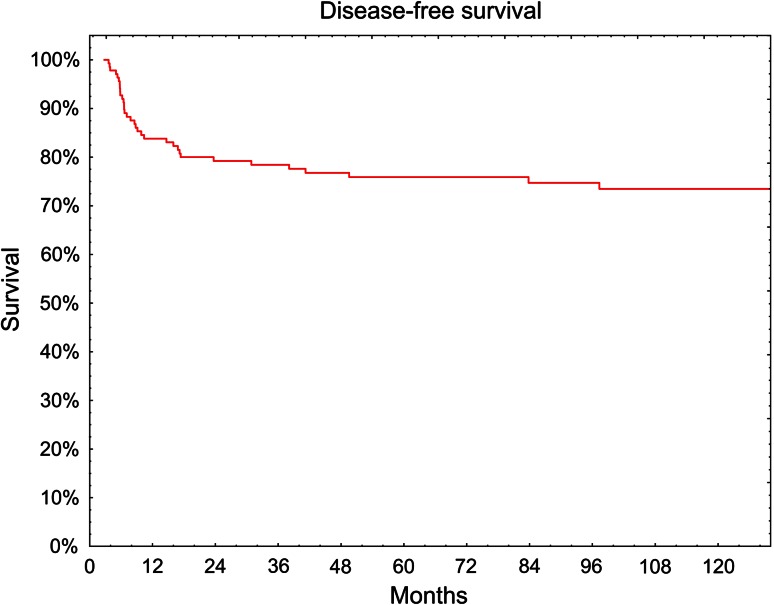
Disease-free survival (Kaplan–Meier curve)

The 5-year overall survival rate was 59 % (Fig. 1). Without evidence of tumor recurrence 5 years survived 104 of 138 patients (76 %) (Fig. 2). Of the 34 patients with reported failure, in 28 (82 %)—locoregional recurrence, in 5 (15 %)—distant failure and in one case local recurrence simultaneously with distant metastases were observed.

Univariate analysis showed that pTNM stage, surgical margins status, pharyngeal invasion, patient age and time between surgery and radiotherapy have significant impact on patient prognosis (Tables 1, 2, 3). Multivariate analysis confirmed prognostic significance of the first three parameters (Table 4).

**Table 1:** The results according to the patient’s characteristics

Characteristics	*n*	DFS5 (%)	*p*
Gender			
Female	6	83	
Male	132	76	0.2689
Age (year)			
≤57	69	80	
>57	69	71	0.0042
Karnofsky performance status (%)			
≤70	25	82	
>70	113	74	0.4640
Haematocrit (%)			
≤41	55	77	
>41	55	72	0.8862
Unknown	28		
Hemoglobin (g/dl)			
≤13	58	77	
>13	47	64	0.851
Unknown	33		

Univariate analysis

**Table 2:** The results according to the tumor’s characteristic

Characteristics	*n*	DFS5 (%)	*p*
pT			
T3	40	92	
T4	98	69	0.0002
Subglottic extension			
Yes	79	76	
No	59	76	0.9092
Pharyngeal invasion			
Yes	29	59	
No	109	80	0.0349
Tracheal invasion			
Yes	10	56	
No	128	77	0.0649
Degree of differentiation			
Well differentiated	36	74	
Moderately differentiated	47	71	
Poorly differentiated	12	80	0.5881
Unknown	43		

Univariate analysis

**Table 3:** The results according to the treatment characteristics

Characteristics	*n*	DFS5 (%)	*p*
Surgical margin			
R0	51	82	
R1	83	72	
R2	4	67	0.0130
Interval between surgery and irradiation (day)			
≤56	70	80	
>56	68	71	0.0250
Overall radiotherapy time (day)			
≤42	76	74	
>42	62	78	0.5190
Total dose (Gy)			
<60	19	78	
≥60	119	61	0.0655

Univariate analysis

**Table 4:** Multivariate analysis (Cox’s model)

Characteristics	Hazard ratio	*p*
pT	2.45	0.00001
Macroscopic disease at surgical margin	2.31	0.0006
Pharyngeal invasion	2.15	0.0041

## Discussion

Detailed analysis of our material suggests that the characteristics of LALC patients postoperatively irradiated at Oncology Centre in Krakow are similar to previously presented literature. Most patients are in the sixth and seventh decade of life, the male-to-female ratio is over 20, the most common site is supraglottic and glottic larynx, pT4 tumors represent 71 % of the presented group [12, 15–17]. In our material macroscopically positive surgical margins were noticed only in four patients, in most cases (60 %) they were positive microscopically. According to the literature this percentage varies from 20 to 70 % [12, 17, 18, 25, 26]. In our study group, as in other papers, low and intermediate grade of tumor differentiation were most common. However, it should be emphasized that in every third patient tumor grading was not specified [11, 12, 20].

In the discussed series of 138 patients DFS5 was 76 %. Our results are similar to those presented by other authors (Table 5). However, it should be underlined that in some of the cited studies the authors described overall survival rates while our own evaluation criterion concerned disease-free survival.

**Table 5:** Postoperative irradiation in locally advanced stage laryngeal cancer

Author	No. of patients	Five-year survival (%)
Kligerman 1995 [25]	76	63
Wieczorek 2002 [12]	90	75
Layland 2005 [10]	56	75
Maillard 2005 [22]	166	69
Milecki 2005 [15]	236	70
Hinerman 2006 [17]	67	92
Present series	138	76

Treatment results

In the presented series the strongest determinant of patient prognosis is pT stage assessed according to the pathological classification. The treatment failure risk in pT4 patients is nearly two and a half times higher in comparison to the cases classified as pT3 (HR = 2,453). DFS5 were 69 and 92 %, respectively (*p* = 0.0002). There is no unequivocal confirmation of relationship between higher pT stage and poor prognosis in LALC patients. Lack of the correlation could be explained by a large heterogeneity of the compared patient groups. It relates to the tumor differentiation, evaluation method of local tumor extension (TNM vs. pTNM) and finally diverse therapeutic procedures for the primary lesion surgery and adjuvant radiotherapy. Nguyen**-**Tan et al. [21] showed a very strong correlation between pT stage and survival rates, OS5 for pT3 and pT4 were 54 and 38 %, respectively (*p* = 0.04). In the study by Yilmaz et al. [23] risk of locoregional failure in T3 and T4 patients were 2.19 and 2.54 times higher compared to T1 stage. Maillard et al. [22] noticed three times higher risk of local failure for the involvement of the whole larynx. On the contrary, Akman et al. [18] showed no predictive value for cT stage. The appropriate OS5 for cT3 and cT4 patients was 65 and 58 % (NS). Similarly Vlantis et al. observed the lack of significant differences in survival rates in relation to the clinical TNM classification. In a group of 50 patients, DFS5 for T3 and T4 was 70 vs 68 % [24]. The same conclusion was obtained by Kligerman et al. [25].

In the discussed study, the surgical margins status is the treatment factor with significant prognostic importance. DFS5 for patients with negative surgical margins (R0) was 82 %, compared to 72 % for a microscopically positive margins (R1) (*p* = 0.013). In a series of 111 patients irradiated postoperatively at the Radiotherapy Department of Pomeranian Medical University, 3-year survival for R0 and R1 was 61 and 41 %, respectively (*p* = 0.005) [26]. The potential prognostic role of radical surgery is emphasized by Hinerman et al., who reported five-year locoregional control (LRC5) of 56 and 89 % (*p* = 0.075) for negative and positive surgical margins, respectively [17]. This is confirmed by the experience of the University Clinic in Vienna. LRC5 for the histologically radical and non-radical surgery in a group of 72 patients, was 58 and 34 %, respectively (*p* = 0.02). The authors noticed a very low efficacy of postoperative irradiation in 82 cases with macroscopically positive surgical margins, where the LRC5 was achieved only in 14 % of patients. In the light of these results, R2 margin seems very doubtful indication for adjuvant radiotherapy [27].

Another parameter characterizing tumor, which prognostic significance was confirmed by the multivariate analysis, was the pharyngeal infiltration. It is connected to DFS5 reduction from 80 to 59 % (*p* = 0.0349). Similar findings were demonstrated by Kligerman`s study on 76 patients, of which 58 were postoperatively irradiated. The exolaryngeal tumor extension, including pharyngeal invasion, increased locoregional recurrence rate from 20 to 39 % (*p* = 0.07) and significantly decreases the DFS5 (*p* = 0.05) [25].

There are conflicting results regarding the prognostic role of the LALC patients` age irradiated postoperatively. The majority of these authors took 55–60 years as a cut-off range. Akman et al. found that five-year overall survival rate (OS5) for patients age groups ≤57 years (*n* = 127) and >57 years (*n* = 126) were 75 and 57 %, respectively. The observed difference was statistically significant, both in univariate (*p* = 0.0008) and in multivariate analysis (*p* = 0.0001) [18]. The prognostic role of age was also emphasized by Vlantis et al. The risk of death in patients aged over 60 years was nearly eight times higher (HR = 7.85) as compared to the younger patients [24]. A different opinion, negating prognostic value of this factor for DFS5 was presented by Nguyen et al. [28] (RR 1.12; CI 0.50–2.51). Interesting study, the aim of which was to determine the probability of distant failure as a function of patient age was conducted by Idasiak et al. Five-year metastases-free survival was assumed as the evaluation criterion. In age subgroups <45 years (*n* = 31), 46–55 years (*n* = 80), 56–65 years (*n* = 119) and >65 years (*n* = 37), it was 85, 81, 84 and 82 % (*p* = 0.707), respectively [29]. Among other Polish investigators, neither Wieczorek nor Sas-Korczyńska confirmed the prognostic significance of patient age for treatment efficacy [11, 12].

In our material, there was no significant effect of performance status (Karnofsky scale) on the treatment results. A similar opinion was presented by Wieczorek [12]. Also Cortesina et al. [19], who used ECOG performance status classification, showed no prognostic role of this parameter.

In each case of a medical procedure including a postoperative radiotherapy, it is important to determine the optimal time for irradiation implementation. It seems that waiting time for radiotherapy (WTR) prolongation may lead to progression of subclinical disease remaining after surgery. In particular, it was clearly evident in poorly differentiated tumors. In our material median WTR was 56 days. We assumed it as a discriminatory value. DFS5 for WTR of ≤56 and >56 days was 80 and 71 % (*p* = 0.02508), respectively. However, multivariate analysis did not confirm the statistical significance of the difference in patients’ survival. Researchers from the University Clinic in Izmir clearly demonstrated that prolongation of WTR above 42 days worsened outcomes by lowering OS5 from 76 to 55 %. This was confirmed by uni- and multivariate analysis where appropriate *p* values were 0.0001 and 0.003 [18]. Wieczorek et al. analyzed the prognostic value of WTR for intervals: <32, 32–43, 44–51 and 52–72 days. Five-year overall survival rates were 63, 61, 44 and 43 %, respectively. The death risk for the longest WTR was almost three times higher (RRH = 2.83) than for WTR less than 32 days [12]. Maillard et al. reported a lack of significant correlation between the risk of local recurrence and WTR of 30 days or longer. Similar conclusion was noticed by Sas-Korczyńska who investigated the relationship for the discriminant of 56 days [11, 22]. None of the remaining analyzed radiotherapy characteristics such as a total dose (cut-off value of 60 Gy) and the overall radiotherapy time (cut-off value of 42 days) significantly affected the patient survival, as in other authors’ studies [11, 18, 28, 29].

In our series 47(34 %) patients underwent tracheotomy due to airway obstruction. DFS5 in this group was 70 % and did not differ significantly from the rate obtained in patients undergoing planned tracheostomy performed before laryngectomy (79 %). In the literature, the reported rate of unscheduled tracheotomy ranges from 20 % to 76 [11, 24, 25, 30]. There is no conclusive opinion about the impact of such procedures on the results of postoperative radiotherapy in LALC patients. Herchenhorn et al. showed a very unfavorable prognostic impact of urgent tracheotomy. The authors of the National Cancer Institute in Rio de Janeiro compared the median OS5 in patients who underwent tracheotomy in planned and urgent mode. The corresponding values were: 56 vs 12 months [30]. Investigators of the University Hospital Centre in Reims identified nearly eight times higher local failure risk in patients undergoing unplanned surgery in comparison to scheduled tracheotomy performed during laryngectomy [22]. According to other authors, urgent tracheotomy does not deteriorate patient prognosis [11, 24, 25].

In the discussed series subglottic tumor extension did not significantly affected survival, what was concurrent with the other authors` observations [11, 17, 18]. In our material DFS5 was equal (76 %) for both 79 patients with this characteristic and 59 patients without it. However, it should be emphasized, that subglottic extension was the only indication for postoperative radiotherapy only in 38 % patients. DFS5 for this subgroup was 73 %. Although the patient cohort is too small, to unequivocally determine this parameter as the obligatory indication (in the absence of others risk factors) for postoperative radiotherapy.

Our results did not show prognostic significance of the trachea involvement, which is in line with the literature [17, 18, 23, 26].

In our case series, the grade of tumor differentiation did not significantly affect patients’ disease-free survival. However, the fact that in 31 % of patients this characteristic was not specified, should be taken into account. The results did not allow us to clearly determine the grade of tumor differentiation as an indicator for adjuvant radiotherapy. The literature on this issue is ambiguous. Some authors, such as Wieczorek et al., showed the lack of relationship between the grade of tumor differentiation and treatment outcomes. However, they did not determine that feature in 38 % of cases [12]. Herchenhorn et al. distinguished two separate cohorts: first classified as G1–3 and the second with only G4 cases. The corresponding median cancer-specific survivals were 56 vs 21 months, but the difference in survival was not statistically significant (*p* = 0.460) [30]. Kubrak and colleagues from the Department of Radiotherapy at Pomeranian Medical University have found that the risk of distant metastases increased three times (21 vs 7 %) with an increase in the malignancy index [26].

The hemoglobin concentration (Hb) and haematocrit are parameters that allow to determine the degree of tissues oxygenation, which affects their radiosensitivity. The better oxygenation the higher local control is observed in irradiated patients (particularly for squamous cell carcinoma of the head and neck and uterine cervix). Recently presented results suggest that anemia may worsen the effectiveness of postoperative radiotherapy in LALC patients [15, 21]. Milecki et al. performed retrospective study in a group of 254 patients (pT3-70 %, pT4-30 %). They evaluated the effect of Hb level (during the first days and at the end of radiotherapy) and Hb value decrease exceeding more than 1 g/dl during adjuvant radiotherapy. Univariate analysis did not show any statistically significant correlation between treatment failure and low Hb concentration in the initial phase of irradiation (*p* = 0.35), whereas this correlation was demonstrated in the final phase of radiotherapy (*p* = 0.004). A similar relationship was noticed for the HB level decline of 1 g/dl and more. Five-year locoregional control rates for Hb >13 and ≤13 g/dl were 74 and 58 %, respectively [15]. Nguyen-Tan et al. found that patients with a Hb level above 12.5 g/dL had a better prognosis compared to lower dominant values. OS5 were 58 and 38 % (*p* = 0.0007), respectively [21]. Idasiak et al. failed to demonstrate significant differences in metastasis-free survival for the following Hb values: <13, 13–14 and >14 g/dl. Convergent conclusion for Hb 14 g/dl was drawn by Sas-Korczyńska [11, 29]. In our case series we showed no correlation between the treatment results and limit values amounting to 41 % and 13 g/dl for haematocrit and hemoglobin, respectively.

In the presented series the main cause of failure was locoregional recurrence, which occurred in 28 out of 34 not cured patients (82 %). Distant metastases were reported in 6 patients (15 %) and the most common site was lungs what was consistent with observations of other authors [22, 24, 25, 28].

Severe late radiation toxicity revealed in 3 (2.2 %) patients. Two cases of skin and subcutaneous tissue fibrosis and one of tracheoesophageal fistula were observed. According to the study by Maillard et al. [22], involving 166 patients with LALC, fibrosis was noticed in 11 patients (7 %), tracheoesophageal fistula in two patients (1 %). Nguyen-Tan et al. assessed a group of 223 LALC patients postoperatively irradiated. They reported tracheocutaneous fistula in three cases (1.3 %) and in three esophageal stricture (1.3 %) [21].

Summarizing our data analysis allowed us to identify the independent prognostic factors (pT, surgical margin status, pharyngeal invasion), presence of which can facilitate the decision about patient eligibility for adjuvant irradiation. However, the optimal treatment strategy for pT3-4N0 LALC remains to be defined by multicentre randomized trials.
